# Dysbiosis and Metabolic Disorders of the Microbiota

**DOI:** 10.3390/metabo16080566

**Published:** 2026-08-10

**Authors:** Guozhu Ye

**Affiliations:** Xiamen Key Laboratory of Indoor Air and Health, State Key Laboratory for Ecological Security of Regions and Cities, State Key Laboratory of Advanced Environmental Technology, Institute of Urban Environment, Chinese Academy of Sciences, Xiamen 361021, China; gzye@iue.ac.cn

## 1. Introduction

**Figure 1:**
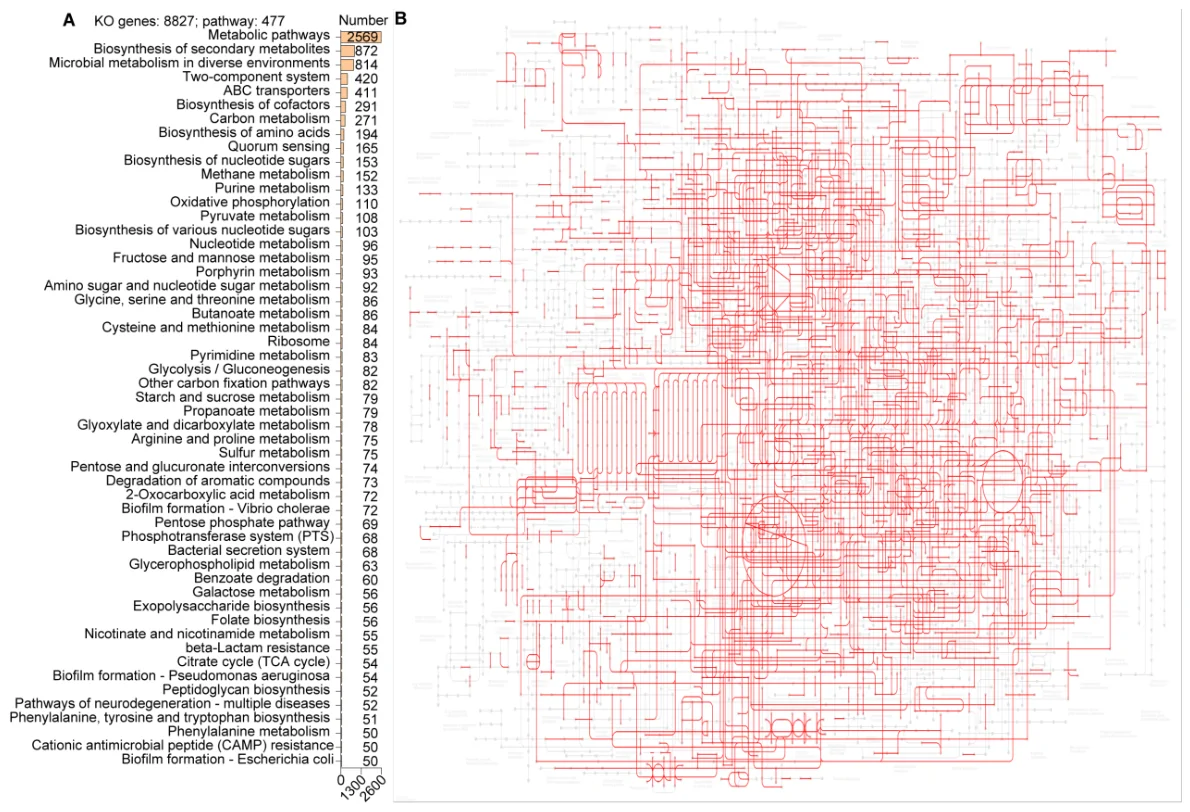
Pathway mapping of KEGG genes from adolescent fecal metagenome. In total, 40 adolescents were enrolled. (**A**) Pathways enriched with at least 50 genes. (**B**) Global visualization of metabolic pathways. Red lines refer to the related genes detected in the sample. Metabolism is involved in most of the pathways.

Microbiota, whether in the gut, skin, or other habitats, has a long history of co-evolution with the host, and plays vital physiological and pathological roles, such as mediating the metabolism, barrier function, immune balance, biological rhythm, and neurobehavior, as well as the occurrence and development of diseases. Microbiota is highly involved in the health of the environment, plants, animals, and humans, and it has become a thriving research field with promising prospects for commercialization. Notably, metabolism is deeply implicated in microbial functions (Figure 1) such as the biosynthesis of bioactive compounds (e.g., neurotransmitters, virulence factors, quorum sensing molecules, and antibiotics). Metabolites are end products of physiological activity, and their changes directly reflect the ultimate response of biological systems to environmental and/or genetic factors [1]. Accordingly, changes in metabolites are closest to the phenotype compared to those in genes and proteins. In recent years, with the rapid development of metabolomics, we are able to more comprehensively decipher microbial functions from the perspective of metabolic outcomes. However, changes in metabolites alone cannot directly provide insights into the initial events and processes of physiological activities. Therefore, it is highly necessary to integrate metabolomics with other molecular-level omics, such as integrating metabolomics with metagenomics and proteomics. This integration allows for the simultaneous analysis of changes in the species/strains, genes, proteins, and metabolites under specific conditions, thereby enabling a multidimensional molecular understanding of the underlying mechanisms, from the initiation to the progression and outcome of molecular events. Given the recent advances in detection technologies and their ability to effectively meet the urgent demands of microbial research, we introduced this Special Issue, aiming to offer useful insights for related studies and technological applications.

## 2. Special Issue Contribution

In total, six articles were included in this Special Issue. Crandall et al. (Contribution 1) found that fungal volatile organic compounds released by *Trichoderma atroviride*, an opportunistic, saprophytic fungal species, had strong repellent effects on mountain pine beetles and slightly affected the feeding activity. Among the volatile organic compounds, phenylethyl alcohol, 2-pentanol, 2-pentanone, and 2-heptanone were suggested as anti-attraction lures for mountain pine beetles in the field test. In another work (Contribution 2), probiotic supplementation (one capsule daily containing *Saccharomyces cerevisiae* var. *boulardii*, *Lactiplantibacillus plantarum* 299v, and octacosanol) was found to improve the morphology of red blood cells and platelets, hemoglobin contents, and chromic status, and decrease the number of activated platelets in obese women.

The microbiome and metabolome were integrated to investigate the dysbiosis and metabolic disorders in response to disease and/or interventions with diets or exercise (Contribution 3–5). In lithogenic diet-induced cholesterol gallstone mice (Contribution 3), lithogenic taxa (such as *Bacteroides stercorirosoris* and *Enterocloster*) were enriched, while protective taxa (e.g., *Akkermansia muciniphila* and CAG-448) were decreased. Meanwhile, serum glycodeoxycholate, N-acetylarginine and long-chain acylcarnitines were increased alongside bile acid and amino acid imbalance. Metabolome and microbiome profiling of rumen fluid from dairy cows (Contribution 4) showed that treatment with a total mixed ration plus galacto-oligosaccharides, essential oil blends, and onion peel increased the metabolism of purine, tyrosine, and tryptophan, and that treatment with a total mixed ration plus mannan-oligosaccharides, essential oil blends, and onion peel activated bile acid biosynthesis and pyrimidine metabolism. In addition, *Macellibacteroides*, *Christensenellaceae*, *Lachnospira*, *Lysinibacillus*, and *Succinivibrionaceae* were increased when the above treatments were performed. Moreover, integrated metabolomics and 16S rRNA gene sequencing (Contribution 5) revealed that decreases in Bacteroidetes, Bacteroidia, Paraprevotella, and Rikenellaceae, as well as an increase in Firmicutes, were observed in post-traumatic osteoarthritis rats with 2-month treadmill walking, and that anthranilic acid and daidzein were increased, while 1-palmitoyllysophosphatidylcholine was decreased after exercise. Finally, gut microbial dysbiosis and metabolic dysfunctions, as well as potential therapeutic targets and strategies, linked to type 2 diabetes, were summarized (Contribution 6).

## 3. Challenges and Opportunities

Due to diverse structures, heterogenous physicochemical property, and the wide concentration range of microbial metabolites, profiling microbial metabolites with a wide and/or deep coverage is still a significant challenge. In addition, bioactive metabolites have important physiological and/or pathological functions, such as quorum sensing molecules and metabolites linked to virulence factors. Therefore, detecting bioactive metabolites with low abundances is a challenging and urgent need. However, methods aimed at detecting highly bioactive metabolites with low abundances could greatly expand and deepen our understanding of microbial functions. In addition, due to the gap between different disciplines, integrating metabolomics data with those from other omics and disciplines is still not effective. Moreover, each method has its limitations. For example, changes in metabolites directly reflect the results of physiological activities, but they do not directly show the causes or processes behind these activities. Accordingly, integrating metabolomics with other omics (e.g., metagenomics and/or proteomics) and/or phenotype data (e.g., diseases, microbial virulence, gut barrier function, and/or some specific molecular pathophysiology) is useful for studying microbial functions [2,3,4,5,6].

The interaction between microbes and their hosts is an important area of research in studying microbial functions. Interactions between metabolites and their receptors (many of which are involved in the two-component system and/or its signaling pathways) are key molecular events in the study of microbe–host interactions, such as responses of the chemoreceptor Tsr to sugars (e.g., fructose, glucose, and mannose), proton motive force, and serine; those of the chemoreceptor Aer to flavin adenine dinucleotide; those of the chemoreceptor TlpC to short-chain organic acids (e.g., acetate, propionate, lactate, and butyrate); and those of the chemoreceptor Tar to aspartate, maltose, and phenol [7,8,9,10,11,12]. When studying metabolite–protein interactions, besides regulating protein and/or metabolite functions at the gene, protein, and metabolite levels, top-down proteomics provides information on intact protein–metabolite complexes and their modifications. Notably, the throughput, coverage, and depth of top-down proteomics are continually improving. With the rapid advancement of techniques like mass spectrometry, detecting microbial proteins, cell envelop components (e.g., lipopolysaccharide, peptidoglycan, lipoarabinomannan, and teichoic acid), and macromolecule-small molecule complexes will be greatly enhanced, thereby promoting research on microbial functions.
